# Clinical characteristics of patients with pneumonia caused by *Klebsiella pneumoniae* in Taiwan and prevalence of antimicrobial-resistant and hypervirulent strains: a retrospective study

**DOI:** 10.1186/s13756-019-0660-x

**Published:** 2020-01-03

**Authors:** Chih-Han Juan, Shih-Yu Fang, Chia-Hsin Chou, Tsung-Ying Tsai, Yi-Tsung Lin

**Affiliations:** 1 Division of Infectious Diseases, Department of Medicine, Taipei Veterans General Hospital, Number 201, Section 2, Shih-Pai Road, Beitou District, Taipei, 11217 Taiwan; 2 Division of Infectious Diseases, Department of Internal Medicine, Puli Branch of Taichung Veterans General Hospital, Nantou, Taiwan; 3 Department of Medicine, School of Medicine, National Yang-Ming University, Taipei, Taiwan; 4 Institute of Emergency and Critical Care Medicine, National Yang-Ming University, Taipei, Taiwan

**Keywords:** Antimicrobial resistance, Capsular type, Hypervirulent strain, Pneumonia, *Klebsiella pneumoniae*

## Abstract

**Background:**

We aimed to compare the clinical characteristics of patients with community-acquired pneumonia (CAP), healthcare-associated pneumonia (HCAP), and hospital-acquired pneumonia (HAP) caused by *Klebsiella pneumoniae* and analyze the antimicrobial resistance and proportion of hypervirluent strains of the microbial isolates.

**Methods:**

We conducted a retrospective study on patients with pneumonia caused by *K. pneumoniae* at the Taipei Veterans General Hospital in Taiwan between January 2014 and December 2016. To analyze the clinical characteristics of these patients, data was extracted from their medical records. *K. pneumoniae* strains were subjected to antimicrobial susceptibility testing, capsular genotyping and detection of the *rmpA* and *rmpA2* genes to identify hypervirulent strains.

**Results:**

We identified 276 patients with pneumonia caused by *K. pneumoniae*, of which 68 (24.6%), 74 (26.8%), and 134 (48.6%) presented with CAP, HCAP, and HAP, respectively. The 28-day mortality was highest in the HAP group (39.6%), followed by the HCAP (29.7%) and CAP (27.9%) groups. The HAP group also featured the highest proportion of multi-drug resistant strains (49.3%), followed by the HCAP (36.5%) and CAP groups (10.3%), while the CAP group had the highest proportion of hypervirulent strains (79.4%), followed by the HCAP (55.4%) and HAP groups (41.0%).

**Conclusion:**

Pneumonia caused by *K. pneumoniae* was associated with a high mortality. Importantly, multi-drug resistant strains were also detected in patients with CAP. Hypervirulent strains were prevalent in all 3 groups of pneumonia patients, even in those with HAP.

## Background

The guidelines of the American Thoracic Society (ATS)/Infectious Diseases Society of America (IDSA) published in 2005 and 2007 classified pneumonia as community-acquired pneumonia (CAP), healthcare-associated pneumonia (HCAP), hospital-acquired pneumonia (HAP), or ventilator-associated pneumonia (VAP) depending on the route of infection [1, 2]. Recently, the ATS/IDSA removed HCAP from the HAP/VAP group in their 2016 guideline update due to the relatively low proportion of multi-drug resistant (MDR) pathogens in this category [3].

*K. pneumoniae* is a major cause of CAP in Asia and is associated with a high mortality in this region, but it rarely causes CAP in Western countries [4–6]. We previously demonstrated that community-onset pneumonia (CAP and HCAP) caused by *K. pneumoniae* was associated with high 28-day mortality (29.7% of patients) and that the nasopharynx may be a reservoir of *K. pneumoniae* [4]. A retrospective cohort study in the United States of America identified *K. pneumoniae* as the second most common gram-negative pathogen responsible for HCAP [7]. *K. pneumoniae* is an important cause of nosocomial pneumonia, which accounts for around 10% HAP and VAP cases [8] and is associated with the rising rate of MDR strains [9].

In the past 3 decades, hypervirulent *K. pneumoniae* (hvKp) strains that cause liver abscess with metastatic septic lesions and other pyogenic abscess have emerged in East Asia [10]. The polysaccharide capsule of *K. pneumoniae* is the most important virulence factor of the pathogen; certain capsular types, such as K1, K2, K5, K20, K54, and K57, are associated with community-onset pyogenic infections [4, 10, 11]. Furthermore, the regulator of the mucoid phenotype genes (*rmpA* and *rmpA2*) regulate extracapsular polysaccharide synthesis, thereby controlling the *K. pneumoniae* mucoid phenotype [12]. There is no consensus on the definition of hvKp strains, but the presence of the virulent capsular types and the expression of the *rmpA/rmpA2* genes are typical features [13]. The diagnostic accuracy of such biomarkers that differentiate hypervirulent from classical *K. pneumoniae* strains has recently been validated [14].

We previously observed that strains with these capsular types are more common in patients with CAP than in those with HCAP [4]. Nonetheless, published studies on the distribution of hypervirulent strains in patients with CAP, HCAP, and HAP caused by *K. pneumoniae* are lacking. We therefore aimed to compare the clinical characteristics of such patients in a geographical region where hvKp is prevalent and assess the proportion of antimicrobial-resistant and hypervirulent strains among the microbial isolates.

## Methods

### Study design and patients

A retrospective study was conducted at Taipei Veterans General Hospital, a tertiary-care teaching hospital in Taiwan, from January 2014 to December 2016. All adult patients (aged > 20 years) consecutively admitted with pneumonia caused by *K. pneumoniae* were included. Patients with polymicrobial infections were excluded, as were patients with incomplete medical records and those diagnosed with liver abscesses with septic lung metastasis, because such abscesses caused by *K. pneumoniae* are prevalent in Taiwan. The study protocol was approved by the institutional review board of Taipei Veterans General Hospital.

Pneumonia was defined based on the Centers of Disease Control and Prevention (CDC) criteria by the presence of lung infiltrate with clinical evidence that the infiltrate was of infectious origin, indicated by onset of fever, purulent sputum, leukocytosis, and decline in oxygenation [15]. The definitions of CAP, HCAP, and HAP followed previously described criteria [1–3]. Briefly, CAP caused by *K. pneumoniae* was defined as *K. pneumoniae*-positive isolates identified upon, or within 48 h of admission in patients who did not fit the criteria for HCAP. HCAP caused by *K. pneumoniae* was diagnosed in patients with *K. pneumoniae*-positive isolates identified upon, or within 48 h of admission, who also met any following criteria [1]: 1) having received intravenous therapy at home or in an outpatient clinic within the last 30 days; 2) having received renal dialysis in a hospital or clinic within the last 30 days; 3) having been hospitalized for 2 or more days within the last 90 days; or 4) having resided in a nursing home or long-term care facility. *K. pneumoniae*-caused pneumonia was considered HAP when *K. pneumoniae*-positive isolates were identified in patients more than 48 h after admission. Patients with *K. pneumoniae*-positive isolates discovered more than 48 h after endotracheal intubation were classified as VAP patients as part of the HAP category.

### Patient data collection

The following clinical data was extracted from the patients’ medical records: demographic characteristics, co-morbidities, immunosuppression, surgeries, invasive procedures or devices, surgical drainage, mechanical ventilation, severity of illness, outcome, and mortality. We defined appropriate empirical antimicrobial therapy as the administration of at least one antimicrobial agent to which the causative pathogen is susceptible within 24 h of the index date of microbiological study at the approved route and dosage. We defined appropriate definite antimicrobial therapy as the administration of at least one antimicrobial agent to which the causative pathogen is susceptible after obtaining the result of the antimicrobial susceptibility test within 24 h at the approved route and dosage. Prior antibiotic exposure was defined as at least 2 days of therapy within 30 days prior to onset of pneumonia. The Acute Physiology and Chronic and Prevention Evaluation (APACHE) II score was used to determine the severity of the illness within 24 h of the onset of pneumonia [16].

### Microbiological analyses

The matrix-assisted laser desorption-ionization time-of-flight mass spectrometry (MALDI-TOF MS, bioMérieux SA, Marcy l’Etoile, France) was used for bacterial identification. The VITEK2 system (bioMérieux, Marcy l’Etoile, France) was used for antimicrobial susceptibility test and the results were interpreted according to the guidelines of the Clinical and Laboratory Standards Institute (CLSI) [17]. MDR *K. pneumoniae* strains were defined as those not susceptible to at least one agent in 3 or more antimicrobial categories [18].

To identify the capsular genotypes of *K .pneumoniae* isolates, we performed *cps* genotyping using the polymerase chain reaction of K-serotype-specific alleles at the *wzy* loci, including serotypes K1, K2, K5, K20, K54, and K57 [11]. The detection of *rmpA* and *rmpA2* genes was performed as described previously [12]. Hypervirulent strains were defined as those had *rmpA* or *rmpA2* genes [14].

### Statistical analyses

Categorical variables were analyzed using the Chi-square test or Fisher’s exact test, and continuous variables using the Student’s *t*-test and Mann-Whitney U test (Wilcoxon rank-sum test). A two-tailed *p* value < 0.05 was considered statistically significant. All statistical analyses were performed using the Statistical Package for the Social Sciences software, version 17.0 (SPSS; IBM, Chicago, IL, USA).

## Results

### Clinical characteristics of pneumonia patients caused by *K. pneumoniae*

During the study period, a total of 276 consecutive patients with monomicrobial *K. pneumoniae*-caused pneumonia was identified. Of these, 68 (24.6%), 74 (26.8%), and 134 (48.6%) cases presented with CAP, HCAP, and HAP, respectively. VAP accounted for 29.1% of HAP patients. The mean age of all patients was 75.71 ± 14.82 years and the patients were predominantly male (*n* = 217, 78.6%). The 28-day mortality rate was 34.1% (94 patients). The overall in-hospital mortality was 46.7% (129 patients).

The clinical patient characteristics are shown in Table 1. Chronic kidney disease was a co-morbidity that was more common in the CAP group than in the HAP group (66.2% versus 45.9%, *p* = 0.015), whereas the frequency of immunosuppression was higher in the HCAP and HAP groups than in the CAP group (25.7% versus 2.9%, *p* < 0.001; 33.6% versus 2.9%, *p* < 0.001, respectively). Similarly, the use of invasive procedures and medical devices was higher in the HCAP and HAP groups than in the CAP group (78.4% versus 54.4%, *p* = 0.002; 88.1% versus 54.4%, *p* < 0.001, respectively).

**Table 1:** Clinical characteristics of pneumonia patients caused by *K. pneumoniae*

Variable	CAP (*n* = 68)	HCAP (*n* = 74)	HAP (*n* = 134)	*p* value		
				CAP vs. HCAP	HCAP vs. HAP	CAP vs. HAP
Demographics						
Age, (Mean ± SD), years	77.13 ± 14.56	77.03 ± 13.85	74.25 ± 15.43	0.736	0.302	0.161
Gender, male	53 (77.9)	62 (83.8)	102 (76.1)	0.375	0.195	0.772
Days of hospitalization prior to culture, median (IQR), days	0.0 (0.0–0.0)	0.0 (0.0–0.0)	12.0 (5.8–22.3)	N/A	< 0.001	< 0.001
Underlying disease						
Malignancy	26 (38.2)	32 (43.2)	51 (38.1)	0.544	0.465	0.981
Diabetes mellitus	20 (29.4)	33 (44.6)	51 (38.1)	0.062	0.358	0.224
Chronic kidney disease	45 (66.2)	34 (45.9)	75 (56.0)	0.015	0.166	0.163
Hemodialysis	0 (0.0)	3 (4.1)	8 (6.0)	0.246	0.750	0.053
Congestive heart failure	5 (7.4)	14 (18.9)	26 (19.4)	0.043	0.932	0.025
Liver cirrhosis	4 (5.9)	2 (2.7)	8 (6.0)	0.426	0.500	1.000
Cerebral vascular disease	12 (17.6)	24 (32.4)	31 (23.1)	0.043	0.145	0.368
Chronic obstructive lung disease	10 (14.7)	14 (18.9)	16 (11.9)	0.503	0.170	0.579
Collagen vascular disease	0 (0.0)	2 (2.7)	10 (7.5)	0.497	0.220	0.018
Transplantation	1 (1.5)	1 (1.4)	3 (2.2)	1.000	1.000	1.000
Immunosuppression^a^	2 (2.9)	19 (25.7)	45 (33.6)	< 0.001	0.237	< 0.001
Invasive procedures and devices at onset of pneumonia	37 (54.4)	58 (78.4)	118 (88.1)	0.002	0.064	< 0.001
Central venous catheter	4 (5.9)	12 (16.2)	49 (36.6)	0.065	0.002	< 0.001
Nasogastric/Nasojejunal tube	26 (38.2)	43 (58.1)	95 (70.9)	0.018	0.062	< 0.001
Urinary catheter	18 (26.5)	28 (37.8)	79 (59.0)	0.148	0.004	< 0.001
Endotracheal tube^b^	1 (1.5)	0 (0.0)	39 (29.1)	0.479	< 0.001	< 0.001
Tracheostomy	0 (0.0)	3 (4.1)	8 (6.0)	0.246	0.750	0.053
Surgical drainage	0 (0.0)	1 (1.4)	18 (13.4)	1.000	0.002	< 0.001
Surgery within 2 weeks	1 (1.5)	2 (2.7)	33 (24.6)	1.000	< 0.001	< 0.001
Prior antibiotic exposure						
Any antibiotic	0 (0.0)	22 (29.7)	103 (76.9)	< 0.001	< 0.001	< 0.001
1st or 2nd generation cephalosporin^c^	0 (0.0)	7 (9.5)	36 (26.9)	0.014	0.003	< 0.001
3rd or 4th generation cephalosporin^d^	0 (0.0)	3 (4.1)	24 (17.9)	0.246	0.004	< 0.001
β-lactam and β-lactamase inhibitor^e^	0 (0.0)	15 (20.3)	59 (44.0)	< 0.001	0.001	< 0.001
Carbapenem^f^	0 (0.0)	4 (5.4)	16 (11.9)	0.121	0.147	0.002
Fluoroquinolone^g^	0 (0.0)	1 (1.4)	19 (14.2)	1.000	0.002	< 0.001
Aminoglycoside^h^	0 (0.0)	1 (1.4)	3 (2.2)	1.000	1.000	0.552
Tigecycline	0 (0.0)	2 (2.7)	9 (6.7)	0.497	0.334	0.030
Glycopeptide^i^	0 (0.0)	3 (4.1)	24 (17.9)	0.246	0.004	< 0.001
Metronidazole	0 (0.0)	1 (1.4)	3 (2.2)	1.000	1.000	0.552
SOFA score, median (IQR)	6.0 (4.0–10.8)	7.5 (5.0–10.0)	6.5 (4.0–10.0)	0.114	0.083	0.835
APACHE II score, median (IQR)	18.0 (12.0–23.0)	23.0 (13.0–26.3)	20.0 (16.0–25.0)	0.003	0.163	0.020

Data are presented as number (%) of patients, unless stated otherwise

*SD* standard deviation, *IQR* interquartile range, *PSI* Pneumonia Severity Index, *APACHE* Acute Physiology And Chronic Health Evaluation, *N/A* not applicable

^a^Immunosuppression was defined as meeting one of the following criteria: neutropenia, use of corticosteroids, or receiving chemotherapy

^b^Endotracheal tube was defined as patient being intubated at the onset of bacteremia

^c^Including cefazolin and cefuroxime

^d^Including cefoperazone, ceftriaxone, cefotaxime, cefepime, and cefpirome

^e^Including amoxicillin/clavulanate, ampicillin/sulbactam, piperacillin/tazobactam, and ticarcillin/clavulanate

^f^Including ertapenem, imipenem, meropenem, and doripenem

^g^Including ciprofloxacin, levofloxacin, and moxifloxacin

^h^Including amikacin, gentamicin and isepamicin

^i^Including vancomycin and teicoplanin

### Clinical outcomes of pneumonia patients caused by *K. pneumoniae*

The proportion of appropriately empirical antimicrobials in patients was similar between the HCAP and CAP groups and was higher in both groups compared to the HAP group (85.1% versus 72.4%, *p* = 0.037; 94.1% versus 72.4%, *p* < 0.001, respectively) (Table 2). Septic shock at onset of pneumonia was more common in the HCAP group than in the HAP group (44.6% versus 24.6%, *p* = 0.003). The 28-day mortality was highest in the HAP group (39.6%), followed by the HCAP (29.7%) and CAP (27.9%) groups. The overall in-hospital mortality was similar between the CAP and HCAP groups (33.8% versus 41.9%, *p* = 0.322), but was lower in the CAP group than in the HAP group (33.8% versus 48.5%, *p* = 0.047) (Table 2).

**Table 2:** Clinical outcomes of pneumonia patients caused by *K. pneumoniae*

Variable	CAP (*n* = 68)	HCAP (*n* = 74)	HAP (*n* = 134)	*p* value		
				CAP vs. HCAP	HCAP vs. HAP	CAP vs. HAP
Appropriate empirical antimicrobial therapy	64 (94.1)	63 (85.1)	97 (72.4)	0.104	0.037	< 0.001
Appropriate definite antimicrobial therapy	61 (89.7)	62 (83.8)	101 (75.4)	0.300	0.158	0.016
Length of stay after pneumonia, median (IQR), days	23.0 (12.5–40.0)	23.5 (10.8–41)	16.0 (6.0–39.3)	0.859	0.126	0.157
Septic shock when pneumonia	20 (29.4)	33 (44.6)	33 (24.6)	0.062	0.003	0.465
Respiratory failure requiring mechanical ventilation	32 (47.1)	27 (36.5)	27 (20.1)	0.202	0.010	0.001
Mortality						
Early mortality (≤48 h)	5 (7.4)	9 (12.2)	20 (14.9)	0.337	0.582	0.122
28-day mortality	19 (27.9)	22 (29.7)	53 (39.6)	0.814	0.158	0.103
In-hospital mortality	33 (33.8)	31 (41.9)	65 (48.5)	0.322	0.360	0.047

Data are presented as number (%) of patients, unless stated otherwise

*IQR* interquartile range, *ICU* intensive care unit

### Microbiological characteristics of clinical *K. pneumoniae* strains

The susceptibility of the isolated *K. pneumoniae* strains to different antimicrobials is shown in Table 3. The proportion of strains with wild-type antibiotic susceptibility (only resistant to ampicillin) was similar between the HCAP and HAP groups (54.1% versus 43.3%, *p* = 0.149), but was lower in both groups compared to the CAP group (54.1% versus 88.2%, *p* < 0.001; 43.3% versus 88.2%, *p* < 0.001, respectively). The proportion of third-generation-cephalosporin-resistant (ceftriaxone or ceftazidime) strains was significantly higher in the HCAP group than in the CAP group (25.7% versus 7.4%, *p* = 0.004), but lower than in the HAP group (25.7% versus 42.5%, *p* = 0.016). MDR strains were more prominent in the HCAP group than in the CAP group (36.5% versus 10.3%, *p* < 0.001), but less frequent than in the HAP group (not statistical significant, 36.5% versus 49.3%, *p* = 0.076) (Table 3).

**Table 3:** Antimicrobial resistance rates of clinical *K. pneumoniae* strains

Variable	CAP (*n* = 68)	HCAP (*n* = 74)	HAP (*n* = 134)	*p* value		
				CAP vs. HCAP	HCAP vs. HAP	CAP vs. HAP
Amikacin	0 (0.0)	0 (0.0)	6 (4.5)	N/A	0.091	0.099
Gentamicin	3 (4.4)	13 (17.6)	22 (16.4)	0.016	0.832	0.013
Cefuroxime	7 (10.3)	27 (36.5)	64 (47.8)	< 0.001	0.117	< 0.001
3rd generation cephalosporin^a^	5 (7.4)	19 (25.7)	57 (42.5)	0.004	0.016	< 0.001
Ciprofloxacin	3 (4.4)	22 (29.7)	52 (38.8)	< 0.001	0.191	< 0.001
Levofloxacin	3 (4.4)	22 (29.7)	51 (38.1)	< 0.001	0.228	< 0.001
Ertapenem	2 (2.9)	11 (14.9)	33 (24.6)	0.018	0.099	< 0.001
Imipenem	0 (0.0)	0 (0.0)	7 (5.2)	N/A	0.052	0.098
Tigecycline	2 (2.9)	1 (1.4)	10 (7.5)	0.607	0.101	0.344
Wild-type antibiotic susceptibility^b^	60 (88.2)	40 (54.1)	58 (43.3)	< 0.001	0.149	< 0.001
Multidrug resistance^c^	7 (10.3)	27 (36.5)	66 (49.3)	< 0.001	0.076	< 0.001

Data are presented as number (%) of isolates resistant to the antibiotic indicated, unless stated otherwise

*N/A* not applicable

^a^Including either ceftriaxone and ceftazidime

^b^Wild-type antibiotic susceptibility was defined in the isolates as susceptibility to all antibiotics except for ampicillin

^c^Multidrug resistance was defined in the isolates as nonsusceptibility to at least one agent in three or more antimicrobial categories

The distribution of the hvKp strains (defined by the presence of *rmpA*/*rmpA2* genes) or capsular types among all *K. pneumoniae* isolates is shown in Table 4. Of all 276 isolated strains, more than half (*n* = 150, 54.3%) were hvKp strains and 131 (47.5%) belonged to one of the six virulent capsular types (K1, K2, K5, K20, K54, and K57). The proportion of hvKp strains was highest in the CAP group (79.4%), followed by the HCAP (55.4%) and HAP (41.0%) groups. Seventeen (43.6%) of the 39 VAP isolates were hvKp strains. Similarly, the proportion of the six virulent capsular types was highest in the CAP group (67.6%), followed by the HCAP (47.3%) and HAP (37.3%) groups. Notably, MDR-hvKp strains (*n* = 14, 5.1%) were similarly distributed between the 3 pneumonia categories [*n* = 2 (2.9%) in the CAP group, *n* = 4 (5.4%) in the HCAP group, and *n* = 8 (6.0%) in the HAP group].

**Table 4:** Comparison of microbiological characteristics of clinical *K. pneumoniae* strains

Microbiological characteristics of isolates	CAP (*n* = 68)	HCAP (*n* = 74)	HAP (*n* = 134)	*p* value		
				CAP vs. HCAP	HCAP vs. HAP	CAP vs. HAP
Capsular type K1	13 (19.1)	8 (10.8)	13 (9.7)	0.164	0.799	0.059
Capsular type K2	14 (20.6)	15 (20.3)	16 (11.9)	0.963	0.106	0.102
Capsular type K1 and K2	27 (39.7)	23 (31.1)	29 (21.6)	0.282	0.132	0.007
Capsular type K1, K2, K5, K20, K54, and K57	46 (67.6)	35 (47.3)	50 (37.3)	0.014	0.161	< 0.001
Presence of plasmid *rmpA*	53 (77.9)	40 (54.1)	53 (39.6)	0.003	0.044	< 0.001
Presence of plasmid *rmpA2*	49 (72.1)	38 (51.4)	52 (38.8)	0.011	0.080	< 0.001
Hvpervirulent *K. pneumoniae*^a^	54 (79.4)	41 (55.4)	55 (41.0)	0.002	0.047	< 0.001
Hvpervirulent *K. pneumoniae* with antimicrobial resistance^b^	3 (4.4)	7 (9.5)	13 (9.7)	0.330	0.955	0.272
Hvpervirulent *K. pneumoniae* with multidrug resistance^c^	2 (2.9)	4 (5.4)	8 (6.0)	0.682	1.000	0.500

^a^Hvpervirulent *K. pneumoniae* is defined as isolates with *rmpA/rmpA2* genes

^b^Antimicrobial resistance is defined as non-susceptibility to at least one antimicrobial agent in addition to ampicillin

^c^Multidrug resistance is defined as non-susceptibility to at least one agent in three or more antimicrobial categories

## Discussion

The current study analyzed the clinical characteristics of patients with *K. pneumoniae*-caused pneumonia in the 3 different pneumonia categories (CAP, HCAP, and HAP), and evaluated the proportion of antimicrobial-resistant and hypervirulent strains. We observed that the mortality was highest in the HAP group, followed by the HCAP and CAP groups. hvKp strains were prevalent in the 3 categories of pneumonia, with the highest proportion in the CAP group. The HAP group had the highest proportion of MDR strains. The proportion of MDR strains in the HCAP group was significantly higher than that in the CAP group, but the proportion of hvKp strains in the HCAP group was significantly lower than that in the CAP group.

Our previous study, conducted from 2012 to 2014, revealed a high 28-day mortality (29.7%) in patients with community-onset pneumonia caused by *K. pneumoniae* that did not differ between the pneumonia types HCAP and CAP (34.1% versus 25.5%, *p* = 0.372) [4]. In the current study, we confirmed a similar 28-day mortality rate in these groups (29.7% versus 27.9%, *p* = 0.814), but we further discovered an even higher mortality of 39.6% in the HAP group. The overall 30-day reported in the literature for hospitalized patients with CAP caused by all pathogens ranges from 4.0 to 18.0% [19, 20]. The mortality rate for patients with HAP due to all pathogens was found to be 13.1% in the United States [21]. We herein once again emphasized the markedly high mortality of *K. pneumoniae*-caused pneumonia in Taiwan, which may be attributed to the fact that hvKp strains are prevalent in this country.

In 2016, the ATS/IDSA guidelines recommended that HCAP should be regarded as a form of CAP, as HCAP patients do not tend to be at a high risk to contract MDR pathogens [3]. In the present study, we focused on pneumonia caused by *K. pneumoniae* and discovered that the proportion of MDR strains in HCAP patients was lower than that in HAP patients (36.5% versus 49.3%, *p* = 0.076), but significantly higher than that in CAP patients (36.5% versus 10.3%, *p* < 0.001). One recent review suggested that the prevalence of CAP caused by MDR *K. pneumoniae* might be related to the consumption of antibiotics by these patients, particularly in Asian countries [22]. In the era of increasing antimicrobial resistance, the validation of predictive scores for different MDR pathogens is warranted to avoid excessive use of broad-spectrum antimicrobials.

hvKp strains are very common in East Asia and usually cause community-acquired pyogenic infections, even in healthy hosts. Classical *K. pneumoniae* strains are typically associated with multi-drug resistance and primarily affect hospitalized patients with comorbidities [23]. In contrast, hvKp strains are usually susceptible to most antimicrobials except for ampicillin. In the present study, we identified the highest proportion of hvKp strains in the CAP group (79.4%), followed by the HCAP (55.4%) and HAP (41.0%) groups. Furthermore, hvKp strains were common in patients with VAP (17/39, 43.6%). Published studies on the proportion of hvKp strains in hospital-acquired infections are scarce. Yan et al. reported that hvKp strains accounted for 28.6% of all *K. pneumoniae* strains that caused VAP in a university hospital in Hunan, China [24]. Another study conducted in China by Liu et al. identified 46.6% of all *K. pneumoniae* strains causing VAP in elderly patients in two tertiary hospitals as hvKp strains [25].

We herein demonstrated for the first time the notably high proportion of hvKp strains causing HAP/VAP outside of China. This observation suggests that hvKp strains have spread from the communities to hospitals in East Asia. We have previously shown that nasal carriage of hvKp strains is common in outpatient patients [4], and HAP/VAP might be an endogenous infection in hospitalized patients. The hvKp strains attacking these vulnerable patients pose a major concern in terms of hospital-acquired infections. Moreover, Liu et al. found that hvKp strains with antimicrobial resistance causing VAP are increasing [25], consistent with the fact that MDR-hvKp strains have emerged in China and are usually fatal due to the limited choice of antimicrobials and their virulence [26, 27]. We have recently reported the high mortality associated with MDR-hvKp infections in Taiwan [28, 29], and we identified 14 MDR-hvKp strains in the current study.

The major limitation of this study is its retrospective design. In addition, our study was conducted in a single tertiary care teaching hospital and the results may therefore not be generalizable due to different patient populations in other hospitals and countries. Furthermore, we did not perform multi-locus sequence typing of *K. pneumoniae* isolates to describe their clonal distribution among the 3 pneumonia groups. Nonetheless, our study describes fort the first time the clinical characteristics of patients with *K. pneumoniae*-caused pneumonia in the 3 pneumonia categories and the microbiological features of these strains.

## Conclusions

Pneumonia caused by *K. pneumoniae* was associated with a high mortality in general and in the HAP group in particular. Notably, we discovered the prevalence of hvKp strains in all 3 pneumonia groups, even in the HAP group (41% of strains in this group). The fact that vulnerable patients were infected with hvKp strains poses a treatment challenge of patients both from the community and in the hospitals in regions where such strains are prevalent. Surveillance should focus on the prevalence of hvKp strains in addition to MDR strains to allow for appropriate management of this disease in the future.

## Data Availability

All materials and data analyzed during this study are contained within the manuscript.

## Abbreviations

CAP: Community-acquired pneumonia
HAP: Hospital-acquired pneumonia
HCAP: Healthcare-associated pneumonia
hvKp: Hypervirulent *Klebsiella pneumoniae*
MDR: Multi-drug resistant
VAP: Ventilator-associated pneumonia
